# Hypothesis: evidence that the *PRS* gene products of *Saccharomyces cerevisiae* support both PRPP synthesis and maintenance of cell wall integrity

**DOI:** 10.1007/s00294-024-01290-w

**Published:** 2024-05-11

**Authors:** Emily Murdoch, Lilian M. Schweizer, Michael Schweizer

**Affiliations:** 1 School of Energy, Geoscience, Infrastructure and Society, Institute of Life and Earth Sciences, Energy, Geoscience, Infrastructure and Society, Riccarton Campus, Edinburgh, EH14 4AS UK; 2 School of Life Sciences, Riccarton Campus, Edinburgh, EH14 4AS UK; 3 School of Engineering and Physical Sciences, Institute of Biological Chemistry, Biophysics and Bioengineering, Heriot Watt University, Riccarton Campus, Edinburgh, EH14 4AS UK

**Keywords:** PRPP synthetase, CWI signalling, Gene duplication, Non-homologous regions, Targeted database screening

## Abstract

The gene products of *PRS1-PRS5* in *Saccharomyces cerevisiae* are responsible for the production of PRPP (5-phospho-D-ribosyl-α-1-pyrophosphate). However, it has been demonstrated that they are also involved in the cell wall integrity (CWI) signalling pathway as shown by protein–protein interactions (PPIs) with, for example Slt2, the MAP kinase of the CWI pathway. The following databases: SGD, BioGRID and Hit Predict, which collate PPIs from various research papers, have been scrutinized for evidence of PPIs between Prs1-Prs5 and components of the CWI pathway. The level of certainty in PPIs was verified by interaction scores available in the Hit Predict database revealing that well-documented interactions correspond with higher interaction scores and can be graded as high confidence interactions based on a score > 0.28, an annotation score ≥ 0.5 and a method-based high confidence score level of  ≥ 0.485. Each of the Prs1-Prs5 polypeptides shows some degree of interaction with the CWI pathway. However, Prs5 has a vital role in the expression of *FKS2* and Rlm1, previously only documented by reporter assay studies. This report emphasizes the importance of investigating interactions using more than one approach since every method has its limitations and the use of different methods, as described herein, provides complementary experimental and statistical data, thereby corroborating PPIs. Since the experimental data described so far are consistent with a link between PRPP synthetase and the CWI pathway, our aim was to demonstrate that these data are also supported by high-throughput bioinformatic analyses promoting our hypothesis that two of the five *PRS*-encoding genes contain information required for the maintenance of CWI by combining data from our targeted approach with relevant, unbiased data from high-throughput analyses.

## Introduction

### 5-Phosphoribosyl-1-α-pyrophosphate (PRPP) synthesis

PRPP is a required building block for the de novo synthesis and salvage pathways of nucleotides; purine, pyrimidine, pyridine (NAD^+^ and NADP^+^) as well the amino acids histidine and tryptophan. PRPP is synthesized via the reaction between ribose 5-phosphate (R5P) and ATP (Hove-Jensen et al. 2017). Given the central role of PRPP in metabolism genes encoding PRPP synthetase have been identified in bacteria, plants, and mammals. The synthesis of PRPP is essential for all free-living organisms but some parasites, e.g., *Trypanosoma* rely on the host metabolism for PRPP production (Ullman and Carter 1997). *PRS* genes are found in both *Saccharomyces cerevisiae* and humans but differ in number and structure (Ugbogu et al. 2022). The human genome contains three PRPS-encoding genes, one of which, *PRPS3*, is expressed only in the testis (Becker 2001). However, in budding yeast, *S. cerevisiae*, there are five unlinked genes, *PRS1–PRS5*, each of which, in theory, is capable of encoding PRPP synthetase (Carter et al. 1997; Hernando et al. 1998; Vavassori et al. 2005c).

### Functions of Prs1-Prs5 in *S. cerevisiae*

Extensive genetic and biochemical analyses have revealed the existence of two multifunctional complexes, Prs1/Prs3 and Prs2/Prs4/Prs5, in addition to the requirement of at least one of the following heterodimers, Prs1/Prs3, Prs2/Prs5 or Prs4/Prs5 (Hernando et al. 1999, 1998) for survival since PRPP-synthesizing capacity is only measurable if one of the above-mentioned heterodimers is present in the cell. Sequencing revealed that *PRS1* and *PRS5* are longer than *PRS2*, *PRS3* or *PRS4* since they contain non-homologous regions, designated NHR1-1, NHR5-1 and NHR5-2, respectively. Furthermore, systematic phenotypic analyses demonstrated that synthetic lethality occurred if *PRS1* or *PRS3* was deleted from strains lacking *PRS5* or strains lacking either *PRS1* or *PRS3* in combination with deletion of *PRS2* and *PRS4*. The importance of the NHRs is underlined since an NHR is present in each of the three heterodimers, Prs1/Prs3, Prs2/Prs5 or Prs4/Prs5, suggesting that they play an essential role in cell viability.

Previous investigation of *PRS* deletant strains has indicated that loss of *PRS1* or *PRS3* gave rise to caffeine sensitivity, a hallmark of impaired CWI (Schneiter et al. 2000). This observation is consistent with one or other of the NHRs being involved in maintaining an intact CWI pathway. Protein–protein interactions (PPIs) exist between Prs proteins and other polypeptides within the yeast cell, specifically polypeptides associated with the CWI signalling pathway. The NHRs are hypothesized to be vital and explain why Prs1 and Prs5 are fundamental in the minimal functional units and for cell viability and growth (Ugbogu et al. 2016a, 2013).

The importance of each Prs protein for the cell’s functioning is not equal but rather through investigation of deletant strains, it has been suggested that Prs1 is likely to be the most important polypeptide of this family. Loss of Prs1 is known to bring about complete abolition of Prs activity and critical impairment of CWI signalling in yeast (Hernando et al. 1999; Ugbogu et al. 2022, 2013). Evidence of CWI signalling impairment has been seen in strains lacking *PRS1* showing phenotypical caffeine sensitivity, temperature sensitivity, α-factor sensitivity, Calcoflour white resistance and release of alkaline phosphatase (Sauvaget et al. 2019; Schneiter et al. 2000; Wang 2005). Prs2 and Prs4 individually lack significant contribution to the necessary functions in cell viability, growth, together with functioning as evidenced by a lack of notable interaction with proteins of the CWI signalling pathway (Hernando et al. 1999; Ugbogu et al. 2016a, 2013). Prs3 is a significant protein in PRPP synthesis, shown through *prs3*∆ strains having a decreased PRPP synthetase activity (Hernando et al. 1999) but also shown that deletion of *PRS3* combined with deletion of *PRS1* or *PRS5* being synthetically lethal. Finally, Prs5 has a functional requirement in yeast physiology since a double deletion of *PRS3* and *PRS5* is synthetically lethal (Hernando et al. 1999, 1998; Sauvaget et al. 2019; Wang et al. 2004). The in-frame insertions of Prs5 (NHR5-1 and NHR5-2) must not be dismissed as they are hypothesized to influence the interactions which Prs5 is reported to have with the CWI signalling pathway transcription factor Rlm1 and *FKS2* expression (Ugbogu et al. 2016a).

### Human PRPS

In humans, *PRPS* gene products have been discovered which show similarities to those found in yeast through their functioning in PRPP synthesis. However, they vary in the number of genes which code for them and their structure. Human PRPP synthetase (hPRPS) is found in three known forms; PRPS1, PRPS2 and PRPS1L1 which are encoded by the respective genes: *PRSP1, PRSP2, PRSP1L1* (*PRPS3*). *PRSP1* and *PRSP2* are X-linked and *PRSP1L1* is located on chromosome 7 (Becker 2001; Becker et al. 1990; de Brouwer et al. 2010; Iizasa et al. 1989; Tatibana et al. 1989; Ugbogu et al. 2022).

In common with yeast Prs gene products, lack of Prs proteins can have detrimental effects on the human body specifically, decreased Prs activity has been linked to neuropathies (Lenherr et al. 2021; Meng et al. 2019; Nishikura et al. 2019; Puusepp et al. 2020; Synofzik et al. 2014). Missense mutations in *PRPS1* have been linked to several diseases of both nonsyndromic and syndromic nature, e.g., Arts Syndrome (Corvino et al. 2018; Song et al. 2012). The gain-of function of hPRPS leads to superactivity of the enzyme resulting in excess amounts of uric acid in the bloodstream, leading to formation of crystals and gouty arthritis (Becker et al. 1988; de Brouwer et al. 2010; Duley et al. 2011; Porrmann et al. 2017).

PPI are an important aspect of all organisms, irrespective of complexity and provide information about cellular function and biological processes within organisms and how specific proteins may alter these processes (Shatnawi 2000). All the interactions within a cell contribute to what is called the interactome (VanderSluis et al. 2018). Yeast Prs protein interactions have been investigated by means of high-throughput and small-scale studies and allow a better understanding of the inner workings of the yeast cell.

PPIs are classed as genetic or physical and can be determined by several methods including, but not limited to, protein-complementation assay (PCA) and yeast two hybrid analysis (Y2H). Genetic PPIs are determined when deletions/mutations occur in two or more genes and generate an unexpected phenotype, e.g., synthetic lethality and negative genetic responses (Costanzo et al. 2016). For the understanding of functional organization of yeast, genetic interactions are a crucial step in the process (van Leeuwen et al. 2017). Physical PPIs are defined as physical molecular docking contact between proteins and should not be mistaken for two proteins ‘bumping’ into one another by chance (de Las Rivas and Fontanillo 2012). A physical PPI is a connection between gene products, whereas genetic interactions are functional relationships between genes.

### Yeast cell wall integrity

The yeast cell wall is made up of 2 layers; an electron-transparent inner layer and an electron-dense outer layer. The inner layer, made up principally of glucan polymers, β-1,3-glucan chains (80–90%), and chitin (1–2%), provides mechanical strength and elasticity of the cell wall, In addition, β-1,6-glucans are linked to manoproteins via GPI anchors Klis et al., 2004).

The outer layer is a lattice of glycoproteins, shielding the plasma membrane from foreign attacking enzymes by limiting the permeability of the cell wall (Gow and Lenardon 2023; Klis et al. 2004, 2002; Orlean 2012). There are four major functions of the cell wall: (1) protection from osmotic shock, (2) protection against environmental and mechanical stress, (3) establishment and maintenance of cell shape, and (4) providing a scaffold for cell-surface proteins. To maintain these functions and the integrity of the cell wall, a mitogen-activated protein kinase (MAPK) signalling cascade is required, called the CWI pathway. The CWI signalling pathway primarily detects and responds to stress on the cell wall which can arise during normal growth conditions or changes in the surrounding environment (Levin 2005). Disruption of signalling causes impairment of CWI and in extreme cases results in cell lysis due to polarized growth. The CWI signalling pathway is known to be activated by the following: elevated temperature, hypotonic shock, impaired cell wall synthesis and chemical agents that induce cell wall stress, e. g. Calcofluor white and caffeine, along with other stimuli (Levin 2011; Ribeiro et al. 2022).

Proteins of the CWI signalling pathway include GTPase, Rho1; Pkh1/Pkh2; Pkc1; Bck1; Mkk1/2; Mpk1/Slt2 and Mlp1; transcription factors, Rlm1, Swi4/6 and Skn7 (Stark 2004). Specifically, Slt2 and Rlm1 of the CWI signalling pathway have been shown to interact with Prs proteins (Ugbogu et al. 2022; Wang et al. 2004). Interactions of Prs1/Mkk1 and Prs5/Pkc1 based on hypotheses have also been demonstrated (Costanzo et al. 2016; Fasolo et al. 2011; Vavassori 2005; Vavassori et al. 2005b). One of the genes which may be upregulated because of the CWI signalling pathway is *FKS2*. The expression of *FKS2* is thought to be influenced by Prs5 (Ugbogu et al 2016a, b). *FKS2* encodes 1,3-β-D-glucan synthase and is regulated by Rho1 and its expression is increased when *FKS1* is unavailable, i. e., under chronic cell wall stress driven by the CWI signalling pathway, generally during heat shock (Heinisch and Rodicio 2018; Jimenez-Gutierrez et al. 2020a; Orlean 2012; Sanz et al. 2022) Interestingly, deletion of *PRS5* or NHR5-2 negatively affects both Fks2 and Rlm1 expression (Ugbogu et al. 2016a; Wang et al. 2004). The CWI pathway does not stand alone in yeast metabolism but has crosstalk with other MAPK signalling pathways, e.g., the HOG pathway (Heinisch 2020; Jimenez-Gutierrez et al. 2020b), calcineurin signalling (Fuchs and Mylonakis 2009) and interactions of the TOR and PKA signalling pathways (Plank 2022).

The following paper explores and scrutinizes the published data which stands to provide evidence that the Prs gene products; Prs1, Prs2, Prs3, Prs4 and Prs5, not only interact with each other but are also involved in the maintenance of the CWI signalling pathway (Kleineidam et al. 2009; Ugbogu et al. 2022; Vavassori et al. 2005a). PPIs between Prs gene products have been examined thoroughly; however, supporting evidence for CWI signalling pathway interactions with Prs proteins is relatively uncharted territory. To address the hypothesis, raw data from high-throughput and small-scale studies was accessed and used to statistically compare and understand discrepancies found between databases and small-scale studies. High-throughput methods are those which generate large amounts of data, in a fast manner using ‘omics’ technologies allowing entire interactomes to be generated in one research study (Blankenburg et al. 2009). Small-scale methodologies in the case of PPIs are those which consider only a few proteins of importance and their interactions, these studies commonly produce more reliable results because of the specificity of them.

## Materials and methods

PPI data deemed relevant to the hypothesis in question were extracted and collated for consideration of the numerical data which provided evidence for such interactions. SGD (https://www.yeastgenome.org/) and BioGRID^4.4^ (https://thebiogrid.org/) were both initially examined for Prs/Prs interactions. Both databases contain an abundance of data on genetic and physical interactions, enabling discovery of relationships between genes and sequences within yeast*.* SGD and BioGRID databases were systematically scrutinized by selecting interactions of yeast Prs with other Prs proteins and seemingly unrelated proteins. Attention was paid to the number of studies which had identified the interaction, whether it was high-throughput or small-scale and the method by which the interaction had been determined. Careful consideration was given to ensure interactions which were selected had a high degree of confidence (e.g., more than 2 studies conducted on the interaction, reliable source, reliable data which was confirmed by another study).

Following identification of Prs/Prs interactions, CWI signalling pathway proteins were identified. The previous two databases were reanalyzed for interactions of Prs proteins with CWI signalling proteins (i.e., Prs1/Slt2, Prs3/Rlm1). All interactions of Prs with other proteins which were uninvolved in the CWI signalling cascade were disregarded and deemed irrelevant to the hypothesis apart from Nuf2, a kinetochore-associated protein (Suzuki et al. 2016).

Extraction of high-throughput interaction data which were deemed relevant and had research to support them, were assessed by exploring the methods and analysis described in the studies. Supplementary material in the form of raw data (where available) was downloaded from research papers. The publications of high-through put analyses which used protein-fragment complementation assays (PCA) (Tarassov et al. 2008) and published data by (Ito et al. 2001a, b) which is based on Y2H analysis were selected as notable studies due to their experimental methods, number of interactions they identified and, specifically, which interactions they identified as seen in their supplementary materials.

Copious amounts of small-scale data have been published on both Prs/Prs interactions and interactions of Prs with CWI proteins. The published data was extracted for both types of interactions and displayed in tabular form (Tables 1 and 2). Interactions of Prs proteins and various elements of the CWI signalling pathway (Slt2, Rlm1, and FKS2) are based on data obtained using β-galactosidase activity reporter assays (Sauvaget et al. 2019; Ugbogu et al. 2022, 2016a, 2013; Wang et al. 2004).

**Table 1:** Prs Protein Protein Interactions

Protein	Interactor	G/P	High	Small
Prs1	Prs2	P	✓	✓
Prs1	Prs3**	G		✓
		P	✓	✓
Prs1	Prs4	P	✓	
Prs1	Prs5*	G		✓
		P	✓	
Prs2	Prs4	P	✓	
Prs2	Prs5	P	✓	✓
Prs3	Prs5*	G		✓
		P	✓	
Prs4	Prs5	P	✓	✓

Prs/Prs interactions identified from SGD and BioGRID databases. Interactions are deemed either genetic or physical interactions. Some have been identified via both high throughput and small-scale methods whereas for others it is just one method of identification. Genetic Interactions (G), Physical Interaction (P)

*Indicates synthetic lethality. Meaning when both proteins are deleted, the cell is no longer viable

**Synthetic lethality but only under exposure to heat (37 °C). Small = Small-scale. High = High throughput

**Table 2:** PPIs between Prs and other proteins. Primarily those of the CWI Signalling Pathway Proteins

Protein	Interactor	G/P	High	Small
Prs1	Mkk1	P	✓	
Prs1	Slt2	P		✓
Prs2	Slt2	P		✓
Prs3	Slt2	G		✓
		P		✓
Prs4	Slt2	P		✓
Prs1	Rlm1	P		✓
Prs3	Rlm1	P		✓
Prs5	Rlm1	P		✓
Prs1	Nuf2*	P		✓
Prs2	Nuf2*	P	✓	✓
Prs3	Nuf2*	P		✓
Prs4	Nuf2*	P		✓
Prs5	Nuf2*	P		✓
Prs5	Pkc1	NG	✓	
Prs5	Fks2	-		✓

Interactions have been extracted from databases: SGD and BioGRID. PPIs are either genetic (G) or physical (P). Determination of interaction is either high throughput or small-scale. Small = Small-scale. High = High-throughput

NG Negative Genetic

*Protein not involved in the CWI signalling pathway

**Fig. 1:**
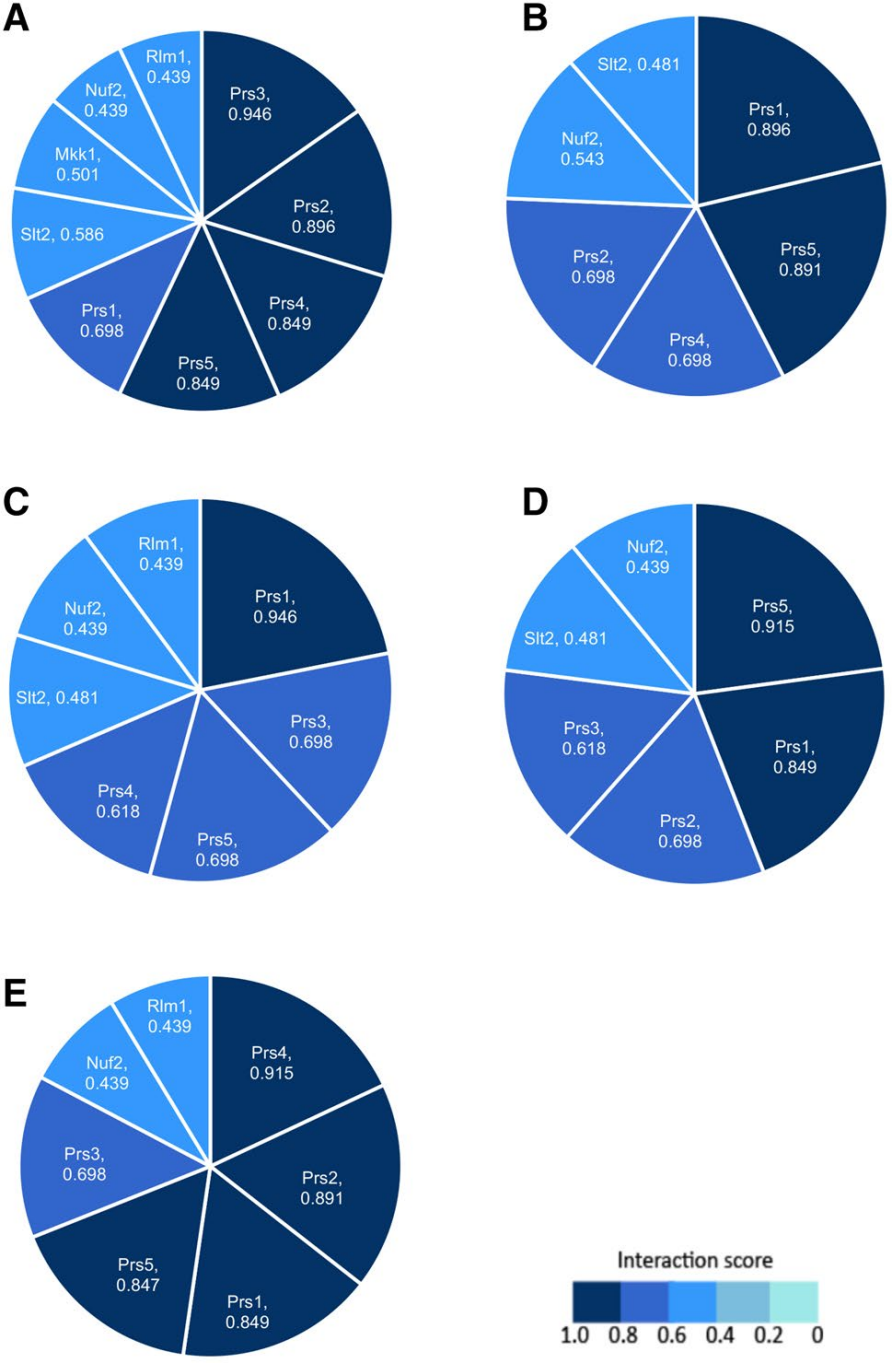
Physical Prs protein-protein Interactions (PPIs) and Prs/CWI pathway protein interactions identified from Hit Predict with corresponding interaction scores. (**A**) Prs1 PPIs, (**B**) Prs2 PPIs, (**C**) Prs3 PPIs, (**D**) Prs4 PPIs and (**E**) Prs5 PPIs. Segment size varies and is dependent on the number of proteins the Prs protein interacts with, and the computed interaction score. The size of the segment is directly proportional to the interaction. A greater interaction score = a larger segment. An interaction score > 0.28 is deemed high confidence. Data were extracted from Hit Predict (http://www.hitpredict.org/index.html)

To determine any further physical interactions which are not documented in SGD and BioGRID, the database Hit Predict (http://www.hitpredict.org/) (Lopez and Nakai 2015) and STRING (https://string-db.org/) (Szklarczyk et al. 2023) were used. These databases have identified interactions for yeast, as well as other organisms. Hit Predict evaluates only physical interactions collated from the following databases; IntAct (https://www.ebi.ac.uk/intact/) (Orchard et al. 2014), HPRD (http://www.hprd.org/; Peri et al. 2003; Prasad et al. 2009), DIP (https://dip.doe-mbi.ucla.edu) (Xenarios et al. 2002) and MINT (https://mint.bio.uniroma2.it) (Licata et al. 2012) and not otherwise identified consisting of calculated interaction scores for all physical PPIs. The interaction scores corresponding to each PPI documented from SGD, BioGRID and Hit Predict were recorded and converted into pie charts for visual representation of how many identified proteins interact with Prs proteins and the score corresponding to each PPI (cf. Figure 1).

The interaction scores are determined based on the arithmetic mean of the annotation-based and the method-based scores. The annotation-based score is a measure based on the likelihood of the interactions occurring taking into consideration genomic features of the interaction whereas the method-based score is determined experimentally. Method-based score considers: the number of publications, method of interaction identification and the interaction type (association, physical association, and direct interaction). Further to an interaction score, the database grades each interaction with a confidence level of either high or low. To be graded a high confidence interaction they must have an annotation score ≥ 0.5, a method-based score of ≥ 0.485 and a combined interaction score > 0.28.

## Results

### Identification of Prs/Prs interactions

Data on protein interactions from SGD, BioGRID and Hit Predict were extracted and used to analyze methods of determining interactions and understanding discrepancies between studies. PPIs have been identified in both high-throughput studies and small-scale studies of Prs polypeptides (Table 1). Prs1 was shown to interact with Prs2-Prs5 as identified by a combination of genetic and physical PPIs. Prs2 interacts physically with Prs1, Prs4 and Prs5. Prs3 interacts both genetically and physically with Prs1 and Prs5. Prs4 was found to interact physically with Prs1, Prs2, and Prs5. Along with Prs1, Prs5 is the only other protein to interact with all proteins, Prs1-Prs4. The following genetic interactions were identified as synthetically lethal: Prs1/Prs3 (at 37 °C), Prs1/Prs5 and Prs3/Prs5. Interactions of Prs proteins with themselves, such as Prs1/Prs1 are possible; however, these data have been excluded because they are well-researched and understood (https://www.yeastgenome.org/). All the interactions shown in Table 1 are based on interaction scores in Fig. 1.

### Prs proteins interact with other proteins, in particular, with CWI signalling pathway proteins

Prs proteins are shown to be associated with activation of the CWI signalling pathway in response to stimuli (e. g., elevated temperature, chemicals, caffeine etc.). Table 2 shows Prs proteins interacting with proteins of the CWI signalling pathway. The MAP kinase of the CWI pathway, Slt2, is confirmed to interact with Prs1-Prs4. Prs3/Slt2 has also been established as a genetic interaction on two accounts, synthetic lethality, and dosage rescue (Binley et al. 1999; Hernando et al. 1999). The transcription factor Rlm1 is recognized to interact physically with Prs1, Prs3 and Prs5. Prs1-Prs5 are found to interact with Nuf2, a kinetochore protein. Prs2/Nuf2 is the only Nuf2 interaction which has been confirmed through both high-throughput and small-scale methods (Sauvaget et al. 2019; Uetz et al. 2000). Nuf2 strongly interacts with all Prs polypeptides except for Prs4. Despite Nuf2 not being directly involved in the CWI pathway these interactions suggest support of Prs protein interactions other than those associated with the CWI pathway (Sauvaget et al. 2019).

Pkc1, the upstream kinase of the MAP kinase cascade of the CWI signalling pathway, and Prs5 display negative genetic interaction. Pkc1 is part of a family of enzymes involved in controlling protein function by phosphorylation of functional elements of several other proteins (Kamada et al. 1995). A synthetic genetic array (SGA) analysis was used by Costanzo et al. (2016) to automate the combinatorial construction of defined mutants and allows quantitative analysis of genetic interactions. This approach uncovered negative genetic interaction between Prs5/Pkc1 alleles. A negative genetic interaction refers to the case where a double mutant displays a fitness defect that is more extreme than expected (Costanzo et al. 2016). The most severe case of this is synthetic lethality, where two genes, each with a mutation, individually cause a minimal phenotype but when combined in the same cell result in cell death. The study determined an SGA score and *p* value for each Prs5/Pkc1 allele interaction and used these scores to determine if each interaction was significant. An SGA score was significant if < −0.12 for negative genetic interactions and a *p* value < 0.05. Six combinations of *PKC1* alleles and *PRS5* were tested for interaction (https://www.yeastgenome.org/locus/S000000201/interaction). All six combinations returned significant SGA scores < −0.12 and with *p* values < 0.05 (data not shown) (Costanzo et al. 2016).

Upon activation by Bck1, Mkk1 phosphorylates the downstream target, Slt2 (Fuchs and Mylonakis 2009; Levin 2011; Ugbogu et al. 2022). High-throughput analysis by Fasolo et al. (2011) determined 1023 interactions in yeast via protein microarrays, one interaction being between Prs1 and Mkk1 (Table 2). The interaction between Prs1/Mkk1 is identified as a reconstituted physical interaction (Fasolo et al. 2011). As described in Fig. 1, the physical interaction was deemed to be a high confidence interaction with a score of 0.501. Prior to this study, it had been postulated in a small-scale study of the interactions of Prs1-Prs4 with Mkk1 and/or Mkk2. However, when Prs1-Prs4 were tested for β-galactosidase activity, adenine and histidine prototrophy, only β-galactosidase activity was observed, albeit only in one orientation for Mkk1 (Vavassori 2005).

Hit Predict and STRING provide quantified interactions for physical Prs/Prs interactions and Prs/CWI pathway protein interactions, enabling an interaction score to be produced. The score considers how the interaction was identified, whether in a large- or small-scale study, the number of publications which identified it, and the features of the proteins involved. The shading in the pie chart (Fig. 1) corresponds to the interaction score for each interaction where the darkest shading implies an interaction score of 0.8–1.0 and the lightest shading, a score of 0–0.2. For Prs/Prs interactions, the highest score of 0.946 was for Prs1/Prs3, the lowest score, 0.618, was for Prs3/Prs4. For CWI signalling pathway protein interactions with Prs, the most abundant was 0.586 for Prs1/Slt2. The smallest interaction score, 0.439 was calculated for several interactions of Prs with non-Prs proteins, viz. Rlm1/Prs1, Rlm1/Prs3, Rlm1/Prs5, Prs1/Nuf2, Prs3/Nuf2, Prs4/Nuf2, Prs5/Nuf2.

Hit Predict correlates with the physical interactions identified in Table 1 and Table 2 with one exception: identification of interaction Prs3/Prs4 where it was not identified in the SGD and BioGRID databases. However, according to Hit Predict Prs3/Prs4 was said to have an annotation score of 1.0 and a method score of 0.38 that combined gives rise to an interaction score of 0.618.

The research reported in Tarassov et al. (2008) employed a PCA procedure to detect the stated interactions (Table 3). The PCA procedure operates by selecting two proteins of interest which are fused to complementary fragments of a reporter protein. A protein is said to interact when the proteins come together causing the reporter protein fragments to ‘join’ and restore their innate folded structure.

**Table 3:** Prs PPIs determined using PCA

MATa gene name	MATα Gene Name	Intensity	Z-Score	PPV%
PRS1	PRS2	42,721	4.410	99
PRS1	PRS4	46,733	4.567	99
PRS1	PRS5	44,785	4.492	99
PRS2	PRS1	99,173	5.769	99
PRS2	PRS4	61,592	4.952	99
PRS3	PRS5	73,367	4.426	99
PRS4	PRS1	69,048	4.980	99
PRS4	PRS2	79,402	5.214	99
PRS5	PRS3	151,519	6.340	99
PRS5	PRS1	34,990	3.808	99

High throughput genome-wide in vivo screening of *S. cerevisiae* identified Prs PPIs. Intensity, z-score and positive predictive value (PPV%) were calculated for each interaction. Intensity score describes the intensity of colony growth on the plates. A true positive PPI can be inferred if the intensity is above the 20,000 threshold. Z-score represents the number of standard deviation (SD) units by which the sample differs from the population mean. PPV score represents a ratio of true positive interactions over the sum of inferred true and false positives. Data extracted and modified from Tarassov et al. (2008)

The research carried out by Tarassov et al. (2008) used the enzyme dihydrofolate reductase (DHFR) as the reporter protein. Once DHFR is in its native structure its activity is detectable through viable cells expressing the fusion proteins and growth in selective medium (Remy et al. 2007). Each interaction is quantified by an intensity score, z-score and PPV% (positive predictive value) and has been used to identify actual PPIs which are relevant to yeast physiology.

The intensity score for PPIs was determined through obtaining images of the plates and the development of image recognition routines from the Image Processing Toolbox of Matlab (Tarassov et al. 2008). After image analysis of plates, excluding the positive and negative controls, the study reasoned that an intensity score above 20,000 could infer a PPI. The data shows that for PPIs in which Prs proteins were partners, they are all above the threshold assigned by the study and implies they are bona fide PPIs. The highest intensity recorded, 151,519, was for Prs5/Prs3 interaction whereas the lowest intensity, 34,990, was for Prs5/Prs1 (Table 3).

The z-score is derived from the distribution of intensities on a plate. The following formula was used to calculate the z-score(*x*) = (*x* − μ_%_)/σ_%_ where *x,* is the intensity of the colony, *μ*_x_ is the average intensity of the plate and *σ*_x_ is the standard-deviation of the mean. The study states that a positive z-score specifies a larger number of interactions within or between two categories compared to a random network. A negative z-score indicates a smaller number of interactions than expected (Tarassov et al. 2008). The highest z-score identified was for Prs5/Prs3, of 6.340. Each interaction includes a literature-sharing score, whether it is synthetically lethal, IST (interaction sequence tag) hit and an IST hit in the opposite orientation (e.g., Prs4/Prs5 and Prs2/Prs4) (Table 4). The original study collated all data and narrowed down core data to be those interactions with 3 IST hits or more. IST hits are ‘the number of detected interactions and the indicator of reproducibility’ and IST hit in opposite direction is ‘the IST hit number for a combination in which bait and prey are in the reverse direction’. The literature-sharing score is ‘the score concerning co-occurrence of prey and bait in the literature, calculated by the calculation formula’ (Ito et al. 2001a).

**Table 4:** Prs PPIs determined using Y2H analysis

BAIT gene	PREY gene	Literature sharing score	Synthetic lethality (1 or 0)	IST Hit	IST Hit in opposite direction
Prs2	Prs1	38	1	3	0
Prs1	Prs3	46	1	3	0
Prs2	Prs1	38	1	3	0
Prs2	Prs5	41	0	8	50
Prs4	Prs5	39	0	4	0
Prs5	Prs2	41	0	50	8

Y2H analysis was carried out to identify all PPIs within yeast. 6 Prs interactions were identified with varying degrees of occurrence. Included is the literature sharing score, whether the interaction is shown to be synthetically lethal, and the interaction sequence tag (IST) hits number for interactions in the forward and opposite direction. Data extracted and modified from Ito et al. (2001a). https://dbarchive.biosciencedbc.jp/en/yeast-y2h/download.html

It has been well established that double deletants *PRS1/PRS5* and *PRS3*/*PRS5* are synthetically lethal at 30 °C (Hernando et al. 1999, 1998) whereas *PRS1*/*PRS3* is synthetically lethal only at 37 °C as determined by Wang et al. (2004). However, Ito et al. (2001a) have described a synthetically lethal interaction between *PRS1/PRS2* using high-throughput methods (Table 4). Variation in synthetically lethal combinations recorded in databases may be caused by human error in the input of data into databases.

The following interactions were identified in this study; Prs4/Prs5, Prs1/Prs2, Prs2/Prs5, and Prs1/Prs3. Prs1/Prs2 and Prs2/Prs5 were also confirmed in the reverse direction (e. g., Prs2/Prs1). The most substantial literature-sharing score was shown to be for Prs1/Prs3 of 46 (Table 4). The combinations; Prs1/Prs2 and Prs1/Prs3, were identified as synthetically lethal. Prs1/Prs2 has been hypothesized to be synthetically lethal. However, it has been determined in low-throughput assays that deleting *PRS1* and *PRS2* reduced the PRPP synthetase activity to 1.2% of the wild type and increased the doubling time to 4 h in contrast to 1.6 h of the WT (Hernando et al. 1999). The highest IST hit, for both the forward and opposite direction, was identified for Prs5/Prs2.For Prs2/Prs5 the IST hit was 50, and in the opposite direction, Prs2/Prs5, was 8.

## Discussion

Understanding and quantification of PPIs can prove beneficial in understanding the biological processes occurring within an organism and provide a better appreciation of the interactome. The first part of this paper examined Prs/Prs interaction and explored the different methods used to determine these interactions and the variation across high-throughput and small-scale studies. Secondly, the paper addressed the interactions of Prs with CWI signalling pathway proteins, taking into consideration the interaction score. Across both parts of the paper, the use of multiple sources provided confirmation of certain interactions, while others were questioned based on insufficient evidence resulting in a low interaction score, e.g., the interaction of Prs1/Prs2 (Ugbogu et al. 2022).

### Prs/Prs interactions

The databases collate an abundance of interactions which have been identified in individual studies. Most of the data of the three databases were comparable, where Hit Predict proved useful in quantifying the interactions. The extracted interactions (Table 1) and the accompanying interaction scores from Hit Predict (Fig. 1) provide reassurance in the interactions and confidence in the methods used in PPI identification. Most of the Prs/Prs interactions, once quantified by Hit Predict had interaction scores greater than 0.6 which corresponds to high confidence in the interaction. There was only minor variation between databases, specifically only Hit Predict recognized Prs3/Prs4 as an interaction which upon inspection of this interaction has been discovered in a small-scale study using anti-tag coimmunoprecipitation (Betel et al. 2007). Variation between databases may be attributed to some databases requiring update.

It can be assumed that based on the analysis of the Hit Predict data, the more publications which have identified an interaction, the more confidence there can be of its existence. For example, the interaction of Prs1/Prs3 has been identified in many papers, dating back to (Hernando et al. 1999) whereas the interaction between Prs3/Prs4 has been identified only once (Betel et al. 2007).

### High-throughput methods

Tables 3 and 4 shows variation in which proteins were identified depending on the method used. Tarassov et al. (2008) (Table 3) used PCA to screen protein interactions in yeast and successfully identified five Prs/Prs PPIs in the forward and reverse directions. Data from (Ito et al. 2001a) (Table 4) used the Y2H pooling approach method and determined fewer interactions, four interactions with only two being identified in the reverse (Table 4). Notably, the interaction of Prs5/Prs2 in the forward direction has an IST hit of 50, the greatest IST hit for the Prs interactions identified by Ito et al. (2001a). The large IST hit number suggests a high degree of certainty in this reaction which is confirmed since it is calculated to have an interaction score of 0.891, considerably above the 0.28 threshold for a high confidence interaction according to Hit Predict.

Both Y2H and PCA are high-throughput methods and are suitable choices for determining the interactome of yeast. Y2H is known to have the potential for identifying false positives as it is a process taking place in vitro so it lacks a proper representation of the cellular functions. Carrying out Y2H analysis in both directions of vector and proteins investigated, the number of possible false positives can be reduced (Bruckner et al. 2009). Similarly, PCA uses the folding of a reporter protein to determine an interaction between two proteins. The folding of the reporter protein is irreversible and may present interactions of proteins which are not true positives. To eliminate this, the PPV% calculated for each interaction by Tarassov et al. (2008) is 99%, indicating that they are all true positives.

### Prs proteins interact with components of the CWI signalling cascade

The SGA scores which were determined for the interactions of Prs5 with alleles of Pkc1 were all statistically significant, providing strong support for these findings since activation of Pkc1 by the GTPase Rho1 initiates the cascade of the CWI signalling pathway to the downstream proteins. The importance of Pkc1 in activating a signalling cascade responsible for CWI signifies that Prs5 may have a significant impact on the entire signalling cascade.

A yeast reporter strain was transformed with relevant plasmids, resulting in pairwise combinations of *PRS1–PRS5* with *SLT2* and the transformants were tested for β- galactosidase activity as a measure of the interaction of the individual Prs polypeptides with Slt2 showing that the strength of interactions is: Prs3 > Prs1 > Prs4 > Prs2 > Prs5 (Hernando et al. 1999; Wang et al. 2004). The NHR1-1 sequence of Prs1 has been hypothesized to be a vital component of the Prs1 protein. To assess the requirements of NHR1-1 for the interaction of Slt2/Prs1, the relative β-galactosidase activity of Slt2/Prs1 (∆NHR1-1) was reduced by more than 80% consistent with sequences in NHR1-1 contributing to the interaction of Prs1 and Slt2, providing evidence for the importance of NHR1-1 in maintaining CWI. This observation was confirmed by co-immunoprecipitation which illustrated that only when Slt2 is phosphorylated do Prs1 and Slt2 interact although the kinase-dead version of Slt2 still interacts with Prs1 (Ugbogu et al. 2016b). It should be noted that for an interaction to be considered as high confidence it must be > 0.28, although this is not the only criterion. The high confidence grade also considers the annotation- and method-based scores (Fig. 1).

The other interaction of note is Prs3/Slt2 which is both physical and genetic (Table 2) (Binley et al. 1999; Ugbogu et al. 2016a; Wang et al. 2004). The synthetic lethality of a *prs3Δ prs5Δ* strain is due to the loss of the pentameric motif _284_KKCPK_288_ at the C-terminal region of Prs3 as demonstrated by FOA counterselection (Sauvaget et al. 2019). Finally, evidence of Prs5/Slt2 interacting is shown by β-galactosidase activity (Wang et al. 2004).

Further confirmation of Prs polypeptides interacting with other components of the CWI pathway is demonstrated by the interaction of Rlm1 with Prs1, Prs3 and Prs5 with the strength of reporter activity decreasing as follows: Prs5/Rlm1 > Prs1/Rlm1 > Prs3/Rlm1. The identification of these interactions using this method has been quantified by Hit Predict. Prs1/Rlm1, Prs3/Rlm1 and Prs5/Rlm1 are all deemed to have interaction scores of 0.439 (cf. Figure 1), thereby emphasizing that Prs1, Prs3 and Prs5 do interact with the CWI signalling pathway, albeit to varying degrees with Prs5/Rlm1 showing the highest level of β-galactosidase activity (Ugbogu et al. 2016a).

There was a notable amount of interaction observed between Rlm1/Prs5 raising the question: do the three phosphorylation sites of NHR5-2 (Ficarro et al. 2002) impact on Rlm1 expression? (Ugbogu et al. 2016a). These serine phosphorylation sites are located within a cluster of six amino acids at positions S364, S367 and S369. When all three phosphosites were mutated Rlm1 activity was increased by 50% at ambient temperature but was capable of further increase at 37 °C. In contrast, only S364A reduced Rlm1 expression in comparison to the wild type at 30 °C. However, each of the individual mutations showed a temperature-dependent increase in Rlm1 activity with the mutation S369 displaying WT temperature-dependent response whereas the other two mutations, S364A and S367A, supported a temperature-dependent increase in Rlm1 activity, albeit less than that observed in the WT (*p* < 0.05, Tukey HSD test) (Ugbogu et al. 2016a). The high value of β-galactosidase activity for Prs5/Rlm1 can be explained by Prs5 phosphorylation sites where the deletion of certain sites has a detrimental effect on Rlm1 expression (Ugbogu et al. 2016b). This result emphasizes the importance of Prs5 for the CWI signalling pathway to be activated appropriately. In the absence of the identified Prs5 phosphorylation sites, the regulation of Rlm1-regulated genes may be impacted. Rlm1 is also responsible for promoter-induced positive-feedback mechanisms which influence the expression of Rlm1 and Slt2 so that the level of Rlm1 expression may well have a detrimental effect on the CWI signalling pathway (García et al. 2016).

Determination of *FKS2* expression revealed that in contrast to the WT, mutation of the phosphosites in NHR5-2 in Prs5 affects its expression at both 30 °C and 37 °C. It is known that in the absence of *FKS1*, the gene responsible for encoding synthesis of β-1,3-glucan synthase, the paralogous *FKS2* will be upregulated by activation of the transcription factor Swi4/6 (Orlean 2012; Ribeiro et al. 2022). The altered expression of *FKS2,* when the Prs5 phosphorylation sites are mutated, is consistent with a vital role of Prs5 in the CWI signalling pathway. *FKS2* expression at 30 °C remained similar for the individual mutations when compared to the WT with only a slight increase in expression for the triple mutant, S364A, S367A and S369A. However, at 37 °C there was a statistically significant decrease in *FKS2* expression for S364A and the triple mutant S364, S367 and S369 [prs5(479)] (*p* < 0.05) when compared to the WT. *FKS2* expression in mutation S367A was minimally increased at 30 °C in comparison to the WT but was higher than prs5(479) and S364A at 37 °C. *FKS2* expression in S369A showed the highest *FKS2* expression of the three mutations and was similar to the WT at 37 °C (Ugbogu et al. 2016a).

The interaction of Prs proteins and Nuf2 is used to support the theory that Prs3 can act as a transporter protein upon stress to the cell wall (Sauvaget et al. 2019). All the interactions of Prs with Nuf2, except Prs2/Nuf2, were the lowest scores extracted from Hit Predict, however, still above the threshold of 0.28 to be graded a high confidence interaction. Prs2/Nuf2 notably has been confirmed in a high-throughput (Uetz 2002; Uetz et al. 2000; Uetz and Hughes 2000) and a precise small-scale study (Sauvaget et al. 2019) which may explain the large interaction score above 0.543.

This paper has assessed different interactions of Prs with various proteins. The importance of specific interactions has been discovered through appreciation of the investigation methods involved which uncovered these interactions and the impact they have on cell viability. A specific example of how gene duplication (Ehrenreich 2020) followed by acquisition of non-intronic, intervening sequences has led to the concept of an interactome linking synthesis of PRPP with the maintenance of CWI. Variations have been found between data sources which have been scrutinized for reasons as to why such variations occur. Different data sources use different values to quantify interactions and comparison has been attempted where possible to relate these values (e.g., intensity, IST hits, β-galactosidase activity). It can be suggested that where an interaction has only one published source, whether it be a high-throughput, or a small-scale study, further research may be beneficial to improve the reliability of the interaction. The novel hypothesis discussed here is supported, not only from data presented in numerous publications from our laboratory but also in publicly available databases. The data are consistent with the five Prs polypeptides of *S. cerevisiae* being involved in two essential aspects of cellular metabolism, provision of PRPP and maintenance of CWI by virtue of three bifunctional heterodimers Prs1/Prs3, Prs2/Prs5 and Prs4/Prs5. The Prs1 and Prs5 polypeptides, both contain non-identical in-frame insertions permitting them to interact with proteins of the CWI pathway, albeit losing the ability to synthesize PRPP, thus explaining the requirement for the three above-named heterodimers for survival.

## Data Availability

The data discussed have been compiled from peer-reviewed publications and information sourced from relevant open access databases as cited in the text.
